# Graphene-Loaded LiNbO_3_ Directional Coupler: Characteristics and Potential Applications

**DOI:** 10.3390/nano15141116

**Published:** 2025-07-18

**Authors:** Yifan Liu, Fei Lu, Hui Hu, Haoyang Du, Yan Liu, Yao Wei

**Affiliations:** 1 School of Information Science and Engineering, Shandong University, Qingdao 266237, China; yifanliu@mail.sdu.edu.cn; 2 School of Physics, Shandong University, Jinan 250100, China; hhu@sdu.edu.cn (H.H.); haoyang.du@mail.sdu.edu.cn (H.D.); 3 Key Laboratory of Laser and Infrared System, Ministry of Education, Shandong University, Qingdao 266237, China; liuyan2021@mail.sdu.edu.cn; 4 Engineering Management Department, Jinan Puli Water Supply Engineering Co., Ltd., Jinan 250024, China; weiyao0204@126.com

**Keywords:** graphene, LiNbO_3_, coupling efficiency, temperature dependent

## Abstract

This study explores the impact of graphene integration on lithium niobate (LiNbO_3_, LN) ridge waveguides and directional couplers, focusing on coupling efficiency, polarization-dependent light absorption, and temperature sensitivity. Experimental and simulation results reveal that graphene loading significantly alters the effective mode refractive index and enhances waveguide coupling, enabling precise control over light transmission and power distribution. The temperature-dependent behavior of graphene–LN structures demonstrates strong thermal sensitivity, with notable changes in output power ratios between cross and through ports under varying temperatures. These findings highlight the potential of graphene–LN hybrid devices for compact, high-performance photonic circuits and temperature sensing applications. This study provides valuable insights into the design of advanced integrated photonic systems, paving the way for innovations in optical communication, sensing, and quantum technologies.

## 1. Introduction

As a fundamental component of integrated photonics, optical waveguides confine light propagation within a small volume at the micron or sub-micron scale, ensuring high optical density within the waveguide [1,2]. Compared to traditional bulk materials, this enables enhanced optical properties, such as laser performance and nonlinear responses. Ridge waveguides, in contrast to slab waveguides, exhibit low fundamental mode cutoff frequencies, broad bandwidths, low impedance characteristics, and stronger spatial confinement of light propagation, making them particularly suitable for constructing complex photonic devices [3]. In photonic integrated circuits, directional couplers (DCs) are ubiquitous as power splitters/combiners due to their simplicity and ease of fabrication [4,5,6]. A DC is a four-port, symmetric, passive optical structure capable of supporting single-mode transmission and guided-wave propagation in all polarization directions. It operates through evanescent wave coupling between two closely adjacent waveguides, enabling light oscillation between them. DCs are commonly used in ring resonators [7,8], Mach–Zehnder interferometers (MZIs) [9], and optical filters [10,11] for splitting and combining optical power. Evanescent wave coupling is highly sensitive to variations in waveguide geometric parameters, such as the gap between waveguides, the effective mode refractive index (n_*eff*_), and the waveguide cross-section. These parameters form the basis of waveguide device design and are essential for constructing novel integrated photonic chip platforms. One of the focuses of the next generation of photonic integrated devices is to improve the performance of optical waveguides by tuning and improving their structure, thus enabling the development of integrated photonic devices combining miniaturization, high efficiency, and high precision. The introduction of two-dimensional materials into waveguide structures to modulate and optimize the transmission of light is a particularly attractive research topic.

Graphene, with its unique optoelectronic properties such as high electron mobility, zero bandgap, and tunable light absorption, has been demonstrated to have numerous photonic and optoelectronic applications. Due to its two-dimensional nature, graphene can easily be incorporated into integrated circuits or photonic circuits, enabling unique optical effects in integrated microstructures [12,13,14,15]. For example, graphene exhibits only 2.3% absorption at normal incidence, but when loaded onto a waveguide, the absorption can reach up to 100% by increasing the interaction length between transmitted light and graphene. Additionally, due to the different mode distributions in waveguide structures and the varying responses of two-dimensional graphene to different polarizations, graphene exhibits polarization-selective light absorption. These properties have become the foundation for developing graphene-based waveguide structures for photodetector and optical modulation devices [16,17]. Although research on graphene-integrated waveguide devices spans a variety of materials, from semiconductors to optical crystals, the majority of reported studies focus on silicon-based waveguides. These include absorption in graphene–silica or graphene–Si_3_N_4_ waveguide composite structures [13,18], the impact of graphene on the Q-factor of silicon waveguide micro-rings [19], the enhancement of nonlinear effects in silicon photonic crystals by graphene [20], graphene-silicon waveguide optical modulators [15,21], and graphene-silicon waveguide photodetectors [22], etc.

In contrast to the extensive research on graphene–silicon waveguide composite structures, studies on loading graphene onto other waveguide structures are scarce, and potential applications are rarely reported. Lithium niobate (LiNbO_3_, LN) waveguides, which occupy an important position in optical device applications [23,24,25,26], offer unique opportunities when integrated with graphene [27,28,29,30]. The introduction of graphene can modulate the intensity and distribution of the optical field in the waveguide through changes in light absorption. Additionally, due to the anisotropic properties of LN, its inherent polarization characteristics can influence the electrical properties of graphene, as demonstrated in LN-based field-effect transistor research [31].

For two-dimensional graphene loaded onto waveguides, the carrier properties are determined by the Fermi energy. Ideally, intrinsic two-dimensional graphene has its Fermi level at the Dirac point, but the Fermi energy can be modulated by external electric fields [32,33]. As the electric field tunes the Fermi energy, graphene transitions from a low-conductivity dielectric material to a higher-conductivity semimetal. This tunable carrier concentration is particularly significant for LN crystal substrates with strong spontaneous polarization, as the electric field generated by crystal polarization can influence the Fermi level of the loaded graphene. In previous studies [34], we reported that the prism coupling efficiency of a 600 nm thick X-cut LN-on-insulator (LNOI) structure covered with monolayer graphene was significantly enhanced under near-infrared light excitation. We hypothesized that the two-dimensional graphene on the LN substrate might exhibit a carrier concentration significantly higher than that of intrinsic graphene, and the resulting surface plasmon polariton (SPP) could be one reason for the enhanced prism coupling. Additionally, we simulated the optical transmission of graphene-loaded LN strip waveguides to explore their practical optical effects and potential applications. The results indicated that the addition of graphene affects the coupling period and optical power distribution in the waveguide. This finding could have significant implications for the integration and efficiency of LN waveguide devices. To experimentally verify our simulation results, this study focuses on a composite structure of LNOI ridge waveguides loaded with two-dimensional graphene, using LN directional couplers based on ridge waveguides as functional devices. By comparing simulation designs with experimental results, we aim to investigate the performance changes and potential applications of the coupled waveguides after graphene integration.

## 2. Design and Simulation

**Figure 1:**
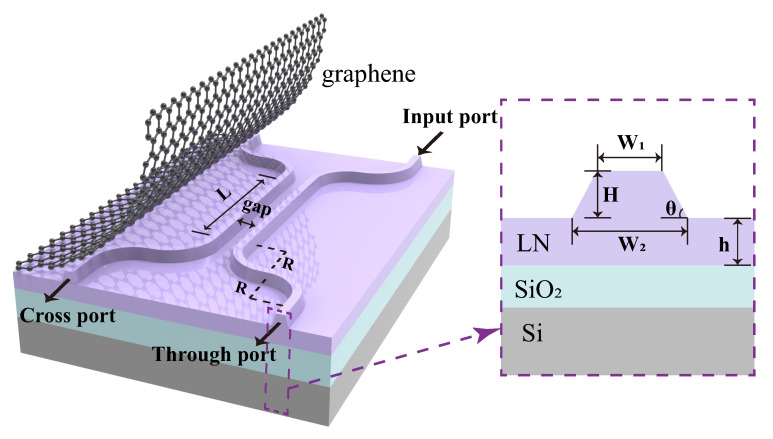
Schematic diagram of a directional coupler structure; purple dotted box illustrates the profile of the ridged waveguide.

The material used for fabricating an LN directional coupler consists of a multilayer structure arranged from top to bottom as LN/SiO_2_/Si, with corresponding layer thicknesses of 600 nm, 2 µm, and 500 µm, respectively. The LN thin film is X-cut, and the designed optical waveguide transmission direction is along the y-axis. The directional coupler structure includes straight waveguides (input/output ports), bent waveguides, and coupled waveguides (two adjacent straight waveguides). A schematic diagram of the waveguide and the corresponding design parameters are shown in Figure 1. Each directional coupler contains two coupled waveguides guided by two 90° bent waveguides. Incident light enters from one input port, propagates through the first 90° bent waveguide, and enters the coupled straight waveguide. Due to the close spacing between two coupled straight waveguides, transmitted light couples the optical power to the other waveguide with the help of evanescent waves in the medium between two waveguides. Ultimately, the remaining optical power in the original waveguide is output at the through port, while the coupled optical power is output at the cross port. Considering the practical fabrication of LN ridge waveguide directional couplers using inductively coupled plasma etching, the cross-sections of waveguides were designed as isosceles trapezoids with an angle of θ = 75°. To ensure single-mode operation of the LN ridge waveguide at a wavelength of 1.5 µm, the finite-difference time-domain algorithm was employed for simulation. The numerical simulations were performed using Lumerical FDTD (2020 R2 version). The optical properties of LN were modeled using the Sellmeier equation, while graphene’s conductivity was calculated through the Kubo model. Perfectly matched layer (PML) boundary conditions were implemented, with the simulation time set to 3000 fs and a maximum mesh step size of 0.02 µm for the override mesh region. We configured the ridge waveguide with an etch depth of H = 300 nm, which is half the thickness of the LN thin film, with the other half being a flat layer with h = 300 nm. Numerical simulations of the individual LNOI waveguide confirmed that single-mode transmission was achieved when the top width W1 = 0.9 µm and the bottom width W2 = 1.2 µm. The bending radius R of the 90° bending waveguide was set to 50 µm to ensure the transmission efficiency of light in the LN ridge waveguide [35].

**Figure 2:**
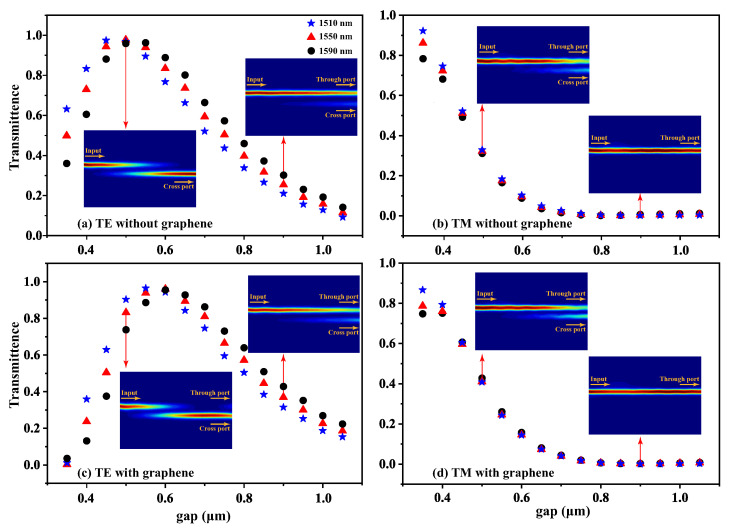
The cross port transmittance varies with the different gap of directional coupler. The (**a**,**b**) LNOI and (**c**,**d**) graphene–LNOI corresponding to TE and TM polarization, respectively. The insets show the power maps at 1550 nm with a gap equal to 0.5 µm or 0.9 µm.

Based on the preliminary determination of the structural parameters of a single waveguide, we conducted simulation calculations to scan the relationship between the transmittance at the cross port and the gap width between two coupled waveguides under different incident light wavelengths. These calculations were performed to determine the range of gap widths that could enable effective coupling between the coupled waveguides, as shown in Figure 2. The inset illustrates the power distribution of light with an incident wavelength of 1550 nm propagating through the coupled waveguides in the directional coupler, providing a visual representation of the power distribution between two waveguides during coupling. The experimental results reveal that the transmittance exhibits a non-monotonic dependence on the waveguide gap, initially increasing before decreasing as the gap widens. The observed smaller spectral variation of TM modes compared to TE modes can be attributed to the distinct electric field distribution characteristic of TM-polarized modes in LN waveguides. Furthermore, variations in the incident wavelength exhibit distinct modulation patterns in the spectral regions preceding and following the peak.

Figure 2a shows that under TE polarization, with a gap of 0.5 µm, the optimal coupling length between two waveguides is L = 40 µm, achieving a maximum cross port transmittance of 0.978 for 1550 nm incident light. Consequently, in the subsequent simulation and fabrication of directional couplers, all coupled waveguide sections were designed with a uniform length of 40 µm. In contrast, under the same coupling length, the transmittance for TM-polarized light is only 0.322, as shown in Figure 2b. To theoretically validate that the loading of graphene affects the optical power distribution in coupled waveguides, we also simulated the optical power distribution in the coupled waveguides of a graphene-loaded directional coupler. Based on our analysis of the carrier concentration in graphene/LN [34], a predicted graphene carrier concentration of 1.19 × 10^13^ cm^−2^ was used in the simulation. The simulation results for different incident polarizations are shown in Figure 2c,d. The results indicate that the introduction of graphene alters the optimal coupling length between the coupled waveguides and causes changes in transmittance. Taking the simulation results in Figure 2c as an example, with a coupled waveguide gap of 0.5 µm and TE polarized light incident, the coupling period between the waveguides is shortened due to graphene loading, resulting in a misalignment of the peak power location of the coupled light and the location of the output ports, which decreases the transmittance at the cross port from about 0.98 at the peak to less than 0.85. Simulations of the waveguide after adjusting the gap width of the coupled waveguide and the polarization state of the incident light also give consistent results.

**Figure 3:**
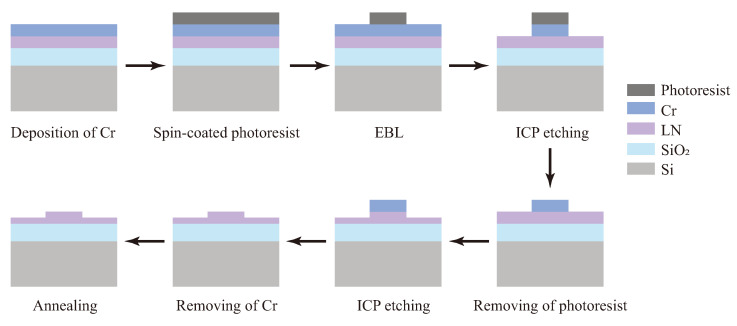
Preparation process of an LN directional coupler.

We propose that graphene integration will modify both the coupling period and optical power distribution in the waveguide system. Experimental verification of this proposition would significantly advance the development of integrated and high-performance LNOI waveguide devices. In order to validate our simulation results for graphene-loaded LN waveguides and to analyze their coupling characteristics, we fabricated two directional couplers with the same ridge waveguide parameters but different gap widths (0.5 µm and 0.9 µm). The manufacturing process of the directional coupler required for the experiment is shown as Figure 3. Initially, an approximately 200 nm thick Cr layer is deposited on the surface of a cleaned LNOI sample to serve as a hard mask, which protects the sample surface. Subsequently, a photoresistant layer (type: HSQ) is spin-coated onto the Cr layer. Following this, electron beam lithography (EBL) and inductively coupled plasma (ICP) etching are employed to pattern the photoresistant and Cr layers, respectively, after which the resistant layer is removed. Thereafter, the desired 300 nm thick LN ridge waveguide is fabricated via additional ICP etching. Finally, the Cr hard mask is removed, residual contaminants adsorbed during the etching process are cleaned, and the sample undergoes annealing treatment. Considering the sensitivity of optical coupling to the distance between waveguides, the closest distance between the coupled waveguides of adjacent directional couplers was set to 500 µm to ensure no optical coupling between them. Additionally, we fabricated ridge straight waveguides with the same parameters for testing the transmission loss of LN ridge waveguides.

## 3. Results and Discussions

**Figure 4:**
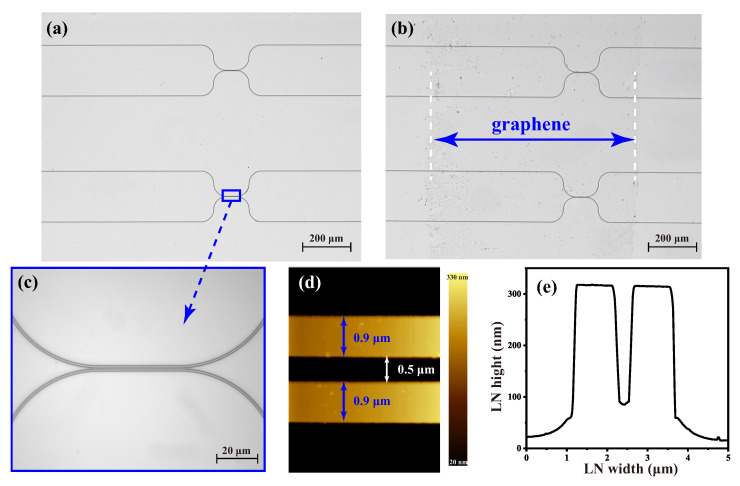
Image of directional couplers observed under an optical microscope (**a**) without graphene and (**b**) with graphene at 10× magnification; (**c**) image of the area in the blue box in (**a**) under an optical microscope at 100× magnification; (**d**) top view and (**e**) cross-sectional height distribution of the coupled waveguides obtained by AFM.

Figure 4a shows an optical microscope image of the coupled waveguides, with the upper directional coupler having a coupled waveguide gap of 0.9 µm and the lower one having a gap of 0.5 µm. The magnified view of the coupled waveguide region of the lower directional coupler (gap = 0.5 µm), along with its top view and cross-sectional view, is presented in Figure 4c and Figure 3d, respectively. The fabricated directional couplers were also tested using AFM to confirm that the actual waveguide parameters met our design requirements, as shown in Figure 4e. The monolayer graphene grown by means of chemical vapor deposition (CVD) was transferred onto the fabricated directional coupler using a wet transfer technique. A graphene flake measuring 0.8 mm × 9 mm was released in deionized water and carefully positioned to ensure complete coverage of the coupled waveguide region. Following extraction from the deionized water, the sample was air-dried at room temperature for 20 min, followed by thermal annealing at 70 °C for 30 min to complete the drying process. Subsequently, the sample was immersed in acetone to completely remove the poly(methyl methacrylate) (PMMA) residual layer. The final transferred graphene, as characterized by optical microscopy, is shown in Figure 4b.

**Figure 5:**
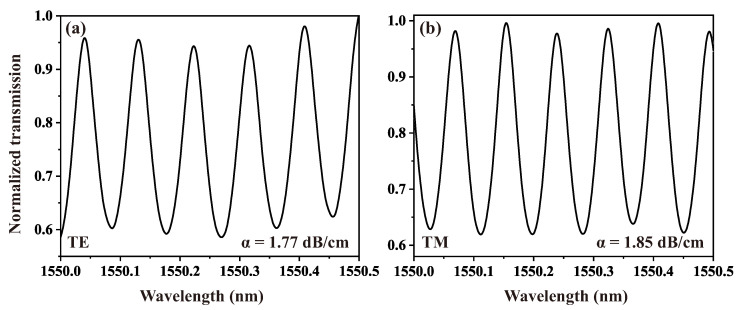
The relationship between the normalized transmission and wavelength of LN ridged waveguides under polarization of (**a**) TE and (**b**) TM.

Due to the inability to directly measure losses in directional couplers using Fabry–Perot methods, we initially characterized the loss of single-mode LN ridge waveguides. These ridge waveguides were designed with identical propagation lengths and cross-sectional parameters to those in the directional couplers, enabling subsequent calculation of the directional couplers’ insertion losses. The polished facet of the straight waveguides form Fabry–Perot cavities, where the output optical intensity exhibits periodic modulation as a function of input wavelength. This interference pattern manifests as characteristic oscillatory behavior in the transmission spectrum. The waveguide propagation loss coefficient α can be extracted from these spectral fringes using Equations (1)–(3), where L represents the length of the straight LN ridge waveguide, R denotes the reflectivity of the LN waveguide polished facet, and $I_{\max}$ and $I_{\min}$ correspond to the maximum and minimum intensities of the transmission spectrum, respectively. The test results, shown in Figure 5a,b, indicate that the transmission losses for TE and TM polarizations in the straight ridge LN waveguides at a wavelength of 1550 nm were 1.77 dB/cm and 1.85 dB/cm, respectively. By comparing the output power of the directional couplers, we also determined the insertion losses of them for TE and TM polarizations to be 2.03 dB/cm and 5.28 dB/cm, respectively. The increased transmission loss is primarily attributed to the radiation loss from the bent waveguides at both ends of the coupled waveguides.

(1) $$\alpha =\frac{4.34}{L}\left(\ln R-\ln R\right)$$

(2) $$\tilde{R}=\frac{1}{k}\left(1-\sqrt{1-k^{2}}\right)$$

(3) $$k=\frac{I_{\max}-I_{\min}}{I_{\max}+I_{\min}}$$

**Figure 6:**
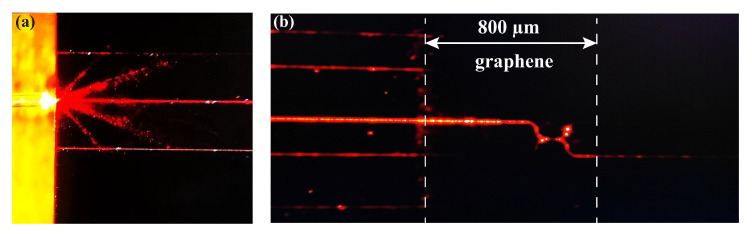
Optical propagation diagram of (**a**) a straight waveguide and (**b**) graphene-loaded coupled waveguides at the wavelength of 633 nm.

For suspended monolayer graphene, its optical absorption in the visible to near-infrared range is a constant given by πα, where the fine-structure constant α = e^2^/(*ℏ*c), where e represents the elementary charge, *ℏ* = h/2π, where h is the Planck constant, and c is the speed of light. Under normal incidence, graphene exhibits an absorption rate of only 2.3%. However, when a layer of graphene is loaded on an LN waveguide, the evanescent wave can continuously interact with the graphene at the interface between the LN waveguide and the graphene, significantly enhancing graphene’s light absorption. This results in a noticeable reduction in the output power of the graphene-loaded waveguide, a phenomenon confirmed by our experimental observations. Figure 6 illustrates the attenuation of 633 nm visible light propagating through the waveguide as it passes through the graphene-covered region. The boundaries of graphene can be roughly determined from the scattered light, and they are marked with white dashed lines in Figure 6b. When the transmitted light in the waveguide is switched to 1.5 µm infrared light passing through the 800 µm graphene-loaded waveguide, the insertion loss in TE and TM polarization increases dramatically to 132.1 dB/cm and 169.15 dB/cm, respectively. These values are approximately 65 and 30 times higher than the losses in waveguides without graphene loading. Given that the two closely adjacent coupling waveguides in the directional coupler have a compact length of precisely 40 µm, the insertion loss induced by graphene can be quantitatively evaluated when the graphene coverage is strictly confined to this coupling region. Based on systematic measurements of the insertion loss in pristine LN waveguides and assuming a linear dependence of the insertion loss enhancement on graphene coverage length, we calculate that graphene integration introduces insertion losses of approximately 47 dB/cm and 58 dB/cm for TE and TM polarization, respectively. While these values are significantly higher than those of the LN ridge waveguide, such loss levels are characteristic of graphene-integrated waveguide systems. Considering that the transmission distance of the graphene-loaded LN waveguide is only 40 µm, the impact of the increased insertion loss on the output power of the directional coupler is acceptable. In addition, these results show that graphene has polarization-selective absorption for light transmitted in LN waveguides. Compared with the loss difference between TE- and TM-polarized light in the LN waveguide, which is only 0.08 dB/cm, this difference in the coupled waveguide region loaded with graphene is 11 dB/cm. This means that with a suitable design of the directional coupler, the strong absorption of TM-polarized light by graphene can be exploited so that only TE-polarized light can be effectively output from the directional coupler. This absorption enhancement brought about by loading graphene onto waveguide structures has become a key concept in the development of novel graphene-based photodetectors and sensors. It is conceivable that by designing the optical field distribution in the waveguide and adjusting the graphene loading length along the waveguide transmission direction, it is expected to achieve a selective output of the optical polarization modes in the waveguide, which could enable the possibility of expanding the applications of directional couplers in optical modulation and optical filtering.

**Figure 7:**
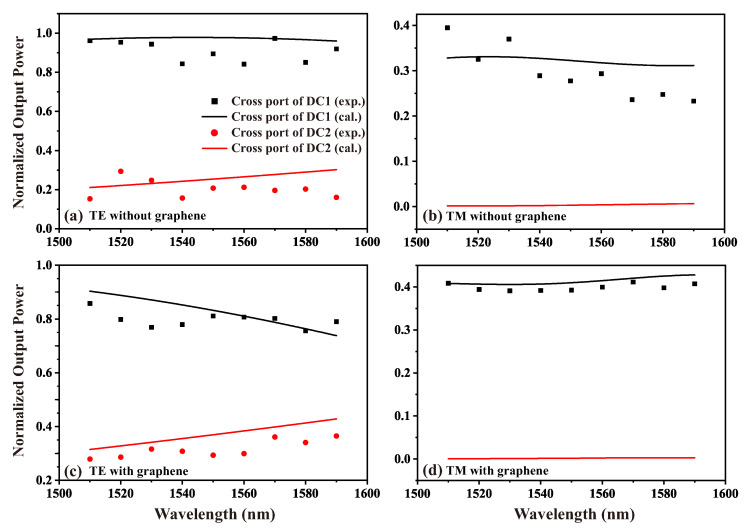
The relationship between the normalized output power and wavelength of the directional coupler. (**a**) TE and (**b**) TM polarization without graphene; (**c**) TE and (**d**) TM polarization with graphene.

To investigate the impact of graphene loading on the optical coupling between coupled waveguides, we measured the output optical power at the cross port of two directional couplers under the same input optical power. We employed peak power normalization to transform absolute physical quantities into relative values, thereby facilitating comparative analysis of the system’s behavior, with the results presented in Figure 7. The discrete points represent the measured normalized output optical power, while the lines correspond to the theoretical simulation results. The black and red colors represent the directional couplers with coupling gaps of 0.5 µm and 0.9 µm, respectively.

Due to manual operations and environmental disturbances during the testing process, the stability of the optical path was affected, introducing some errors into the results. However, as can be seen from Figure 7, the experimental data align well with the simulation results, indicating that the simulation calculations based on our waveguide model reasonably reflect the actual coupling properties between the waveguides. For the directional coupler with a coupling gap of 0.9 µm, the output power at the cross port under TM polarization was too low to be distinguished from noise, making it impossible to obtain experimental results. Therefore, in Figure 7b,d, only simulation results are presented.

Table 1 provides a comparison between experimental and simulated data for the cross port output power ratio of two directional couplers with coupling gaps of 0.5 µm and 0.9 µm at an incident wavelength of 1550 nm. Here, DC and G-DC represent LN ridge waveguides without and with graphene loading, respectively, while 1 and 2 correspond to coupling gaps of 0.5 µm and 0.9 µm.

According to the simulation results, for TE-polarized incident light, when the coupling waveguide gap is 0.5 µm, a coupling length of 40 µm theoretically allows complete coupling of the input light wave to the cross port (99.99%). In actual measurements, due to transmission losses and structural errors, the coupling efficiency is 93.92%. When a single layer of graphene is loaded onto the waveguide, the theoretical simulation predicts a coupling efficiency of 86.30%, while the experimentally measured value is 84.7%. Analysis of the simulated coupling process reveals that the reduction in coupling efficiency at the output port is due to over-coupling. The loading of graphene shortens the distance required for complete coupling between the waveguides, causing some of the light at the cross port to couple back to the through port over the 40 µm interaction length. The agreement between simulated and experimental results also applies to TM polarized light and directional couplers with a gap of 0.9 µm. While theoretically reducing the inter-waveguide gap could decrease the DC length, the minimum achievable gap in practical fabrication is fundamentally constrained by waveguide manufacturing limitations. The graphene-induced reduction in coupling period therefore provides a novel approach for optical device miniaturization. These results show that the loaded graphene results in an enhanced coupling between the two LN coupled waveguides. Comparative analysis reveals that graphene integration reduces the coupling length relative to identical graphene-free LNOI DC, indicating graphene’s ability to actively modulate the electric field distribution between coupled waveguides. This modification enables the precise control of optical transmission and power distribution in LN waveguides, thereby offering a viable approach for designing more compact directional couplers and optical power modulation devices with higher integration density.

The carrier density in graphene is a key parameter that has to be considered when we model the optical properties of graphene/LNOI composite structures. For graphene loaded onto an LN substrate, the carrier distribution and concentration can be affected by a variety of factors, among which the polarization field of the LN crystal and the adsorption of environmental impurities are two major considerations. Given that the carrier concentration and impurity adsorption properties of graphene are sensitive to the ambient temperature, we can analyze the effect of graphene on optical transmission in LN waveguides by studying the temperature effect of LN directional couplers loaded with graphene. To investigate this, we tested the temperature-dependent output from the cross port in the directional coupler over a temperature range from room temperature to 90 °C.

**Figure 8:**
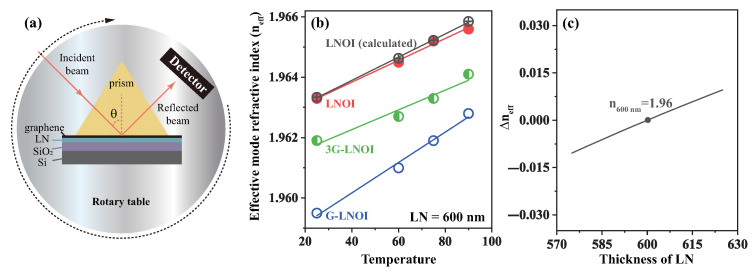
(**a**) Top view of prism-coupled total reflection system when heated. (**b**) The relationship between n_*eff*_ and the temperature. (**c**) The relationship between Δn*_eff_* and the thickness of LN film.

To exclude the contribution of temperature effects on the LN waveguide in the LNOI substrate, we first measured the effective mode refractive index of the LNOI substrate and the same slab waveguides loaded with monolayer graphene (G-LNOI) as a function of temperature. Figure 8a presents a schematic diagram of the prism coupling measurement system. The tested sample comes into contact with the prism base with a precisely controlled air gap maintained between them. A laser beam is incident on the bottom surface of the high-refractive-index prism and is subsequently reflected to a photodetector, with all components (the prism, thin-film sample, and photodetector) mounted on a common rotation stage. The incident angle θ can be varied by rotating the stage. At specific θ values (referred to as mode angles), the evanescent wave tunnels through the air gap from the prism base into the thin film, exciting guided optical modes and consequently causing a sharp dip in the photodetector signal. By measuring the reflected intensity as a function of the incident angle, the effective mode refractive indices (n*_eff_*) for both transverse electric (TE) and transverse magnetic (TM) polarizations can be determined. For controlled heating purposes, we attached a miniature high-temperature ceramic heating element with electrical insulation to the sample substrate. The heating element possesses a resistance of approximately 27 ohms and is powered by an adjustable DC power supply. Thermal energy is transferred to the sample surface through conduction, and after sufficient time, the surface temperature stabilizes at a well-defined value. At this equilibrium state, we consider the graphene layer to have achieved uniform thermal distribution, implying that the charge carrier temperature in graphene equals the temperature of the underlying LN crystal substrate.

The results are shown in Figure 8b, which presents the experimental and calculated values of the effective refractive index for an ∼600 nm thick LN thin-film waveguide as the temperature increases from room temperature to 90 °C. Figure 8c displays the characteristic relationship between n_*eff*_ and thickness for LN thin films under normal conditions, where Δn_*eff*_ denotes the difference in n_*eff*_ between the actual thickness and a 600 nm thick LNOI reference. At room temperature, the n*_eff_* is 1.96 for a 600 nm thick LN film, and Figure 8b confirms that the actual thickness of the LN films used in experiments fluctuates around 600 nm. For pure LNOI thin-film waveguides with a theoretical thickness of 608 nm (black line in Figure 8b), the temperature-dependent experimental values of the effective mode refractive index show excellent agreement with theoretical calculations, confirming that the material and structural parameters used in simulations accurately describe the LNOI waveguide properties. Figure 8b reveals that the G-LNOI waveguide exhibits a greater temperature-dependent increase in the effective mode index compared to the pristine LNOI waveguide. In contrast, the trilayer graphene-integrated waveguide demonstrates nearly identical thermo-optic behavior to the bare LNOI waveguide, indicating that dielectric properties dominate in multilayer graphene systems. Comparative analysis shows that the temperature coefficient of the effective index for monolayer graphene–LNOI waveguides is approximately 1.5 times greater than that of bare waveguides. After subtracting the inherent temperature dependence of the LNOI substrate, this enhancement is unambiguously associated with monolayer graphene integration. The difference due to temperature increase may be related to the presence of hot carriers in graphene. It can be envisioned that as the temperature increases, more hot carriers will be excited in the monolayer graphene material, which in turn will affect its dielectric properties. Since the dielectric properties of monolayer graphene are more susceptible to changes in the concentration of hot carriers and scattering effects, they exhibit a stronger frequency and temperature dependence, especially at high frequencies (e.g., 1550 nm), where the dielectric constant increases significantly.

Before testing and analyzing the directional coupler output power variation with temperature, we need to take into account the effect of the thermal expansion of the LN waveguide with increasing temperature. In order to exclude the effect of thermal expansion of the LN ridge waveguide itself on the structural and transmission characteristics of the waveguide, we calculated the structural parameters of the LN ridge waveguide at a temperature of 90 °C (the highest temperature value in the actual experiment). We constructed a simulation model by comparing these parameters with the effective refractive index measured by prism coupling experiments. It was found that the transmission spectra of the LN waveguide at 90 °C were not significantly different from the results at room temperature. Therefore, we did not consider the effect of the thermal expansion of the LN film on the experimental results in the subsequent tests.

**Figure 9:**
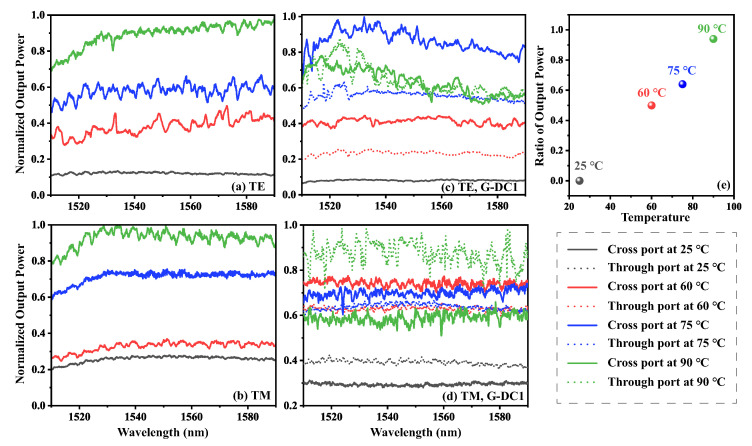
The relationship between the normalized transmission and wavelength at different temperatures under (**a**) TE and (**b**) TM polarization for an LN ridged waveguide and (**c**) TE (**d**) TM polarization for G-DC1. (**e**) The power ratio of the through port to the cross port under various temperature conditions for (**c**).

Figure 9 presents the test results of the output power from a graphene-loaded G-LNOI ridge straight waveguide as well as from the cross port in the directional coupler as a function of temperature. Meanwhile, we implemented precise temperature control by positioning a copper thermal block beneath the sample substrate. Figure 9a,b show the normalized output power of the graphene-loaded G-LNOI ridge straight waveguide under TE and TM polarization conditions, respectively, as the temperature increases. It is clear from these results that the output power of the optical waveguide increases significantly with increasing temperature. The occurrence of this phenomenon is mainly related to two factors: the increase in LN n_*eff*_ with temperature and the thermal effect of graphene. When n_*eff*_ of the LN substrate increases with temperature, the n_*eff*_ difference between the LN and the surrounding material increases, and the constraint of the LN waveguide on the transmitted light is enhanced (which means the enhancement of the optical confinement capability), resulting in an increase in the power of the output light. However, according to the theoretical simulation, the increase in optical power due to the increase in the LN refractive index alone is very limited and cannot reasonably explain the results given in Figure 9a,b. It can be inferred that the addition of graphene and the thermal effect of graphene could play a non-negligible role in increasing the output power of the waveguide.

Since the high loss in graphene-loaded LN waveguides mainly comes from the absorption of light by graphene, which is the main reason for the decrease in the output power, it can be inferred that the increase in the output power may be related to the decrease in the absorption of light by graphene. It has been inferred in the previous analysis that the excitation of hot carriers in graphene leads to an increase in the dielectric coefficient of graphene; however, in addition to this, graphene produces other thermal effects as the temperature increases, including the shift in the Fermi energy levels and the Pauli blocking effect, the free-carrier absorption, and the broadening of the absorption spectrum due to the scattering of carriers. For example, the increase in temperature leads to an increase in carrier concentration, which causes the Fermi energy levels to enter the conduction or valence band, triggering the Pauli blocking effect: low-energy photons that could have been absorbed (e.g., in the infrared region) cannot be absorbed due to the occupation of the target energy level, and the high kinetic energy of hot carriers exacerbates the scattering of carriers–carriers and carriers–phonons, which results in the broadening of the absorption spectrum and smoothes out the overall absorption curve, which also means that the graphene absorption of 1550 nm light will be weakened. The combination of these effects results in a decrease in the absorption of 1550 nm transmitted light by graphene. This is perhaps one plausible component within a complex interplay of mechanisms to explain that why the output power of graphene-loaded LN waveguides increases significantly with temperature.

The enhanced coupling efficiency of the graphene-loaded LN waveguide and the fact that the output power increases significantly with increasing temperature make the power output of the graphene-loaded LN directional coupler highly sensitive to temperature variations, and this sensitivity is especially reflected in the output power ratios of the two output ports of the directional coupler. Figure 9c,d show the changes in the output optical power of the G-DC1 directional coupler at the through and cross ports, respectively, during the heating process. The output power ratios of the cross port and the through port change drastically in the heating range of 27∼90 °C. In the case of TE polarized light with a wavelength of 1540 nm, for example, at room temperature the output light is almost exclusively output from the cross port and the ratio of the output power of the two ports is close to 1:0. When the temperature rises to 60 °C, the ratio is approximately 2:1, at 75 °C, it is close to 3:2, and at 90 °C, it is approximately 1:1. In the case of TM polarized light, the change in the ratio is even more pronounced: at room temperature, the ratio is approximately 1:2, which means that the output power at cross port is only 50% of the output power at through port. However, as the temperature rises to 60 °C, the ratio reverses and the output power of the cross port exceeds that of the through port. As the temperature continues to rise to 90 °C, the ratio reverses again, with the output power of the cross ports exceeding that of the cross ports.

This result shows that for the graphene-loaded LN directional coupler with a special design, the power ratio at the output is sensitive to the temperature change and shows a certain regularity (see Figure 9e), which provides the possibility of determining the temperature change by measuring this ratio. At the same time, due to the large variation of this ratio, the graphene-loaded LN directional coupling can be utilized to realize the temperature sensing function with higher sensitivity and accuracy.

## 4. Conclusions

In summary, this study investigates the effects of graphene loading on the optical coupling properties of lithium niobate (LN) ridge waveguides and directional couplers. Both experimental and simulation results demonstrate that the incorporation of graphene into LN waveguides leads to measurable enhancement in coupling efficiency (e.g., a 3.27% increase in output power for TE polarization in G-DC2 compared to DC2), enabling the precise control of optical transmission and power distribution. Simultaneously, the introduction of graphene significantly increases the loss difference between TE and TM polarizations in LN from 0.08 dB/cm to 11 dB/cm. Furthermore, the temperature-dependent behavior of graphene-loaded LN waveguides exhibits strong thermal sensitivity, with the slope of refractive index variation being approximately 1.5 times greater than that of bare waveguides, rendering these structures promising candidates for temperature sensing applications. This research not only advances the understanding of graphene–LN hybrid photonic devices but also opens up new avenues for developing compact, high-performance integrated photonic circuits with potential applications in optical communication, sensing, and quantum technologies. The ability to tune coupling efficiency and polarization selectivity through graphene integration highlights the potential for innovative designs in next-generation photonic devices.

## Figures and Tables

**Table 1:** The output power of the LN directional coupler at the cross port is obtained via experiments and calculations with or without a graphene covering at a wavelength of 1550 nm.

	TE (%)		TM (%)	
	**Experiment**	**Calculation**	**Experiment**	**Calculation**
DC1	93.92	99.99	20.7	36.13
G-DC1	84.7	86.30	42.0	49.77
DC2	20.23	25.95	-	0.32
G-DC2	23.5	38.18	-	0.23

## Data Availability

The data presented in this study are available on request from the corresponding author.
